# Supplementary material for: The Portfolio Diet and HbA1c in Adults Living with Type 2 Diabetes Mellitus: A Patient-Level Pooled Analysis of Two Randomized Dietary Trials
Source: Nutrients. 2024 Aug 23;16(17):2817. doi: 10.3390/nu16172817 (PMC11397446; doi:10.3390/nu16172817)
Supplement: Supplementary file 1 [file nutrients-16-02817-s001.zip › nutrients-3142697-supplementary.pdf]

Supplement

The Portfolio Diet and HbA1c in adults living with type 2 diabetes mellitus: a patient-level pooled analysis of two randomized dietary trials

Meaghan E Kavanagh, Songhee Back, Victoria Chen, Andrea J Glenn, Gabrielle Viscardi, Zeinab Houshialsadat, John L Sievenpiper, Cyril WC Kendall, David JA Jenkins, and Laura Chiavaroli

Table of Contents

Table S1: Portfolio Diet pillars with the clinical Portfolio Diet Score targets for a 2,000-kcal diet........ 2

Table S2. Pooled raw values for cardiometabolic risk factors at baseline and end (6-months) for control (high fibre dietary advice) and test (low glycemic index dietary advice) groups........ 4

Table S3. Multilinear regression analysis of the associated change in clinical Portfolio Diet Score and cardiovascular risk factors......... 4

Table S4. Mediation analysis, role of dietary glycemic index in the association between the clinical Portfolio Diet Score and HbA1c......... 5

Table S5. Stratified analysis of the association of change in clinical Portfolio Diet Score and change in HbA1c by sex and ethnicity......... 5

Figure S1: Conceptual model for the Portfolio Diet and HbA1c in adults living with T2DM....... 6

Figure S2: Change in the clinical Portfolio Diet Score shown by the control (high fibre dietary advice) and test (low glycemic index dietary advice) groups......... 7

Figure S3: Change in the clinical Portfolio Diet Score by pillars shown by the control (high fibre dietary advice) and test (low glycemic index dietary advice) groups......... 8

Figure S4: Change in clinical-Portfolio Diet Score verses change in the dietary glycemic index. 9

Supplement

Table S1: Portfolio Diet pillars with the clinical Portfolio Diet Score targets for a 2,000-kcal diet.

Portfolio Diet Pillars	Examples of Portfolio Diet foods for 1 c-PDS point
	**2,000 kcal**
**Nuts and seeds, amount/point**	Almonds - 7 nuts
	Walnuts - 4 halves
	Pistachio (shell-free) - 15 nuts
	Peanuts (shell-free) - 8 nuts
	Other nuts - 7 nuts
	Nut or seed butters, all natural - 2 tsp
	Ground flax seeds - 1 tbsp
	Seeds, e.g. pumpkin, sunflower, chia - 1 tbsp
**Plant protein, amount/point**	Soy/Pea beverage (fortified) - 1.25 cups
	Tofu (extra firm or firm) - 1/2 cup
	Tofu (silken, soft) - 2/3 cup
	Tempeh - 1/4 package (50g)
	Edamame (shelled) - 1/2 cup
	Chickpeas (cooked/canned) - 1/2 cup
	Beans (cooked/canned) - 1/2 cup
	Lentils (cooked/canned) - 1/2 cup
	Peas, green - 1 cup
	Veggie burger - 1 patty*
	Veggie sausage patties - 2 patties*
	Veggie meat grounds - 1/3 cup*
	Veggie Chick'n tenders - 3 tenders*
	Soy/pea protein powders** - 1.5 tbsp
**Viscous fibre, amount/point**	100% Psyllium husk powder - 1 tsp
	100% Psyllium husk - 1 tbsp
	Psyllium powder product (e.g. Metamucil) - 2tsp
	PGX® supplement - 2g (2-3 capsules)
	Oat bran (dry) - 2/3 cup
	All-bran Buds with Psyllium -1/2 cup
	Steel cut oats/oatmeal (dry) – 3/4 cup
	Oat bran bread - 1 slice
	Barley (cooked) - 2 cups
	Okra (cooked) - 3 cups

Supplement

	Eggplant (cooked) - 4 cups<br>Persimmons - 2 large<br>Oranges - 2 large<br>Apples - 2 large<br>Berries - 2 cups
**Plant sterols, amount/point**	Plant sterol margarine - 1 tsp<br>Packet - 1/5th packet<br>Plant sterol powder - 1/5 tsp<br>Supplements - 1 capsule§
**High MUFA oils, amount/point**	Extra virgin Olive Oil - 2 tsp<br>Cold press Canola Oil - 2 tsp<br>Cold press Soy Bean Oil - 2 tsp<br>High Oleic Oils - 2 tsp<br>(E.g. High Oleic Sunflower, Safflower or Soy)<br>Canola Oil - 2 tsp<br>Soybean Oil - 2 tsp<br>Avocado - 1/4 fruit

Abbreviations: c-PDS, clinical Portfolio Diet Score; MUFA, monounsaturated fatty acid.

* For plant-based meats, they must come from soy or pulse-based proteins.

**For protein powder, it must come from soy or pulse-based proteins.

§ For Supplements, typically 5 capsules = 2g plant sterols which is the total recommended amount per day.

Supplement

Table S2: Pooled raw values for cardiometabolic risk factors at baseline and end (6-months) for control (high fibre dietary advice) and test (low glycemic index dietary advice) groups

**Outcomes**	**Baseline**		**End (6-months)**		**Change**		**P-value***
	**Control**	**Test**	**Control**	**Test**	**Control**	**Test**	
HbA1c, %	7.06 (0.04)	7.10 (0.05)	6.81 (0.06)	6.49 (0.06)	-0.25 (0.06)	-0.61 (0.05)	<0.0001
LDL-C, mmol/L	2.3 (0.07)	2.4 (0.08)	2.3 (0.07)	2.3 (0.08)	0.01 (0.04)	-0.06 (0.05)	0.31
Non-HDL-C, mmol/L	2.9 (0.07)	3.0 (0.08)	2.9 (0.07)	2.9 (0.08)	-0.01 (0.05)	-0.09 (0.06)	0.30
TC, mmol/L	4.1 (0.08)	4.1 (0.09)	4.1 (0.08)	4.0 (0.09)	-0.01 (0.05)	-0.06 (0.06)	0.45
TG, mmol/L	1.4 (0.08)	1.5 (0.08)	1.3 (0.06)	1.4 (0.11)	-0.07 (0.05)	-0.07 (0.09)	0.96
HDL-C, mmol/L	1.2 (0.02)	1.1 (0.03)	1.2 (0.02)	1.1 (0.02)	0.0001 (0.01)	0.02 (0.01)	0.22
SBP, mmHg	126.4 (1.20)	125.4 (1.15)	123.2 (1.20)	122.4 (0.98)	-3.17 (1.04)	-3.02 (1.06)	0.92
DBP, mmHg	72.0 (0.80)	72.4 (0.81)	70.3 (0.77)	70.6 (0.71)	-1.72 (0.68)	-1.79 (0.65)	0.94

Abbreviations: CI, confidence interval; c-PDS, clinical Portfolio Diet Score, DBP, diastolic blood pressure; HDL-C, high-density lipoprotein cholesterol; LDL-C, low density lipoprotein cholesterol; SBP, systolic blood pressure; TC, total cholesterol; TG, triglycerides.

Values are means (SE). Control n=132, test n=135

*P value for difference in change across test and control group.

Table S3: Multilinear regression analysis of the associated change in clinical Portfolio Diet Score and cardiovascular risk factors.

Cardiovascular risk factor	Beta coefficient* (95% CI)	***P*-value**
LDL-C, mmol/L	-0.03 (-0.05, -0.01)	0.016
Non-HDL-C, mmol/L	-0.04 (-0.06, -0.02)	0.001
TC, mmol/L	-0.04 (-0.06, -0.01)	0.002
TG, mmol/L	-0.04 (-0.07, -0.01)	0.017
HDL-C, mmol/L	0.002 (-0.003, 0.01)	0.40
SBP, mmHg	-0.01 (-0.48, 0.49)	0.98
DBP, mmHg	-0.05 (-0.37, 0.26)	0.72

Abbreviations: CI, confidence interval; c-PDS, clinical Portfolio Diet Score, DBP, diastolic blood pressure; HDL-C, high-density lipoprotein cholesterol; LDL-C, low density lipoprotein cholesterol; SBP, systolic blood pressure; TC, total cholesterol; TG, triglycerides.

*Adjusted for age (continuous), sex (female, male), smoking (yes, no), body mass index (BMI) (continuous), trial (DMGI, DM-MRI), antihyperglycemic medications (yes, no), BP medications (yes, no), lipid lowering medications (yes, no), and prior cardiovascular event (yes, no).

P value for linear regression

To convert total cholesterol, HDL, non-HDL-C, and LDL-C from mmol/L to mg/dL multiply by 38.67.

To convert triglycerides from mmol/L to mg/dL multiply by 88.57.

Supplement

Table S4: Mediation analyses between the clinical Portfolio Diet Score and HbA1c.

Mediator		Beta coefficient**	95% CI	*P*-value
Dietary GI	Model 2*	-0.04	(-0.07, -0.02)	0.001
	Model 2* + dietary GI	-0.02	(-0.05, 0.01)	0.287
Body weight change	Model 2*	-0.04	(-0.07, -0.02)	0.001
	Model 2* + BW	-0.03	(-0.05, 0.01)	0.005

Abbreviations: BW, body weight; CI, confidence interval; GI, glycemic index.

*Model 2 adjusted for age (continuous), sex (female, male), smoking (yes, no), body mass index (BMI) (continuous), trial (DMGI, DM-MRI), antihyperglycemic medications (yes, no), blood pressure medications (yes, no), lipid lowering medications (yes, no), and prior cardiovascular event (yes, no).

**Change per 1 point

P value for linear regression

Table S5: Stratified analysis of the association of change in clinical Portfolio Diet Score and change in HbA1c by sex and ethnicity.

HbA1c (%)	N	Beta-coefficient per 1 point increase in c-PDS (95% CI)	$P_{interaction}$
Sex			0.553
Female	98	-0.05 (-0.09, -0.02)	
Male	169	-0.04 (-0.07, -0.001)	
Ethnicity			0.782
White	180	-0.03 (-0.65, -0.01)	
South Asian	43	-0.02 (-0.08, 0.04)	
East Asian	17	-0.05 (-0.15, 0.05)	
Other*	26	-0.04 (-0.14, 0.07)	

Abbreviations: CI, confidence intervals; c-PDS, clinical Portfolio Diet Score. HbA1c, glycated hemoglobin.

Adjusted for age (continuous), smoking (yes, no), body mass index (BMI) (continuous), trial (DMGI, DM-MRI), antihyperglycemic medications (yes, no), blood pressure medications (yes, no), lipid lowering medications (yes, no), and prior cardiovascular event (yes, no).

*Other includes Black, Native, Hispanic and those who selected “other” ethnicity. Consolidated owing to small numbers.

Supplement

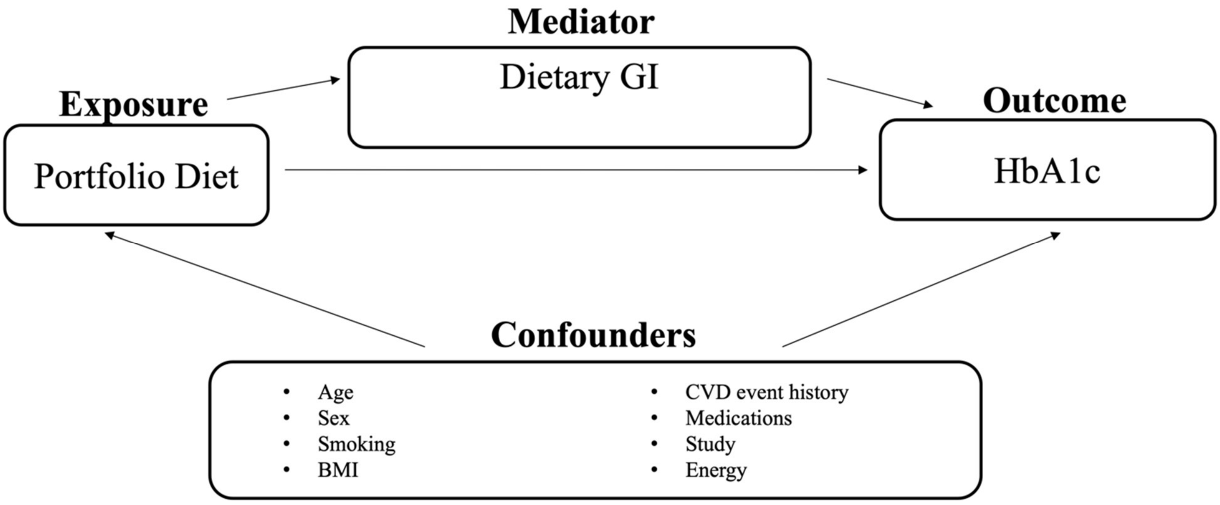

Figure S1: Conceptual model for the Portfolio Diet and HbA1c in adults living with T2DM.

Abbreviations: BMI, body mass index; c-PDS, clinical Portfolio Diet Score; CVD, cardiovascular disease; GI, glycemic index; HbA1c, glycated hemoglobin.

Adjustments included age (continuous), sex (female, male), smoking (yes, no), body mass index (BMI) (continuous), trial (DMGI, DM-MRI), antihyperglycemic medications (yes, no), blood pressure medications (yes, no), lipid lowering medications (yes, no), and prior cardiovascular event (yes, no) as covariates.

Given the c-PDS is adjusted for energy, a separate adjustment for energy was not performed, as this would be an overadjustment.

Dietary GI was predicted to be a mediator for the Portfolio Diet and HbA1c.

Supplement

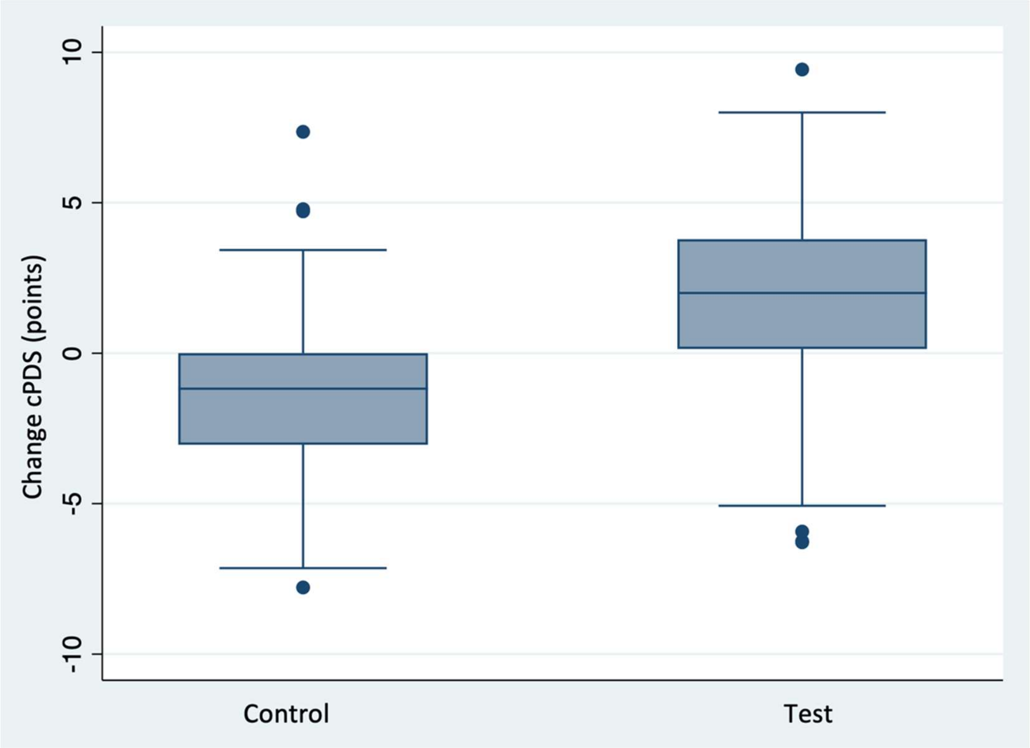

Figure S2: Change in the clinical Portfolio Diet Score shown by the control (high fibre dietary advice) and test (low glycemic index dietary advice) groups.

Abbreviations: c-PDS, clinical Portfolio Diet Score.

The center line in each box represents the 50th percentile (median change). The bottom of each box represents the 25th percentile of c-PDS and the top of each box represents the 75th percentile of c-PDS. The whiskers represent the upper and lower extremes with dots showing outliers.

The 6-month c-PDS for those in the control groups was 2.6 points (10%), ranging from 0 to 13 points (0% to 52%) and for those in the test groups was 5.8 points (23%), ranging from 0.5 to 18.3 points (2% to 74%).
The mean change in c-PDS from baseline to 6-months for those in the control groups was -1.5 points, ranging from -7.8 to 7.4 points. The mean change for those in the test groups was 1.7 points, ranging from -6.3 to 9.4 points.

Supplement

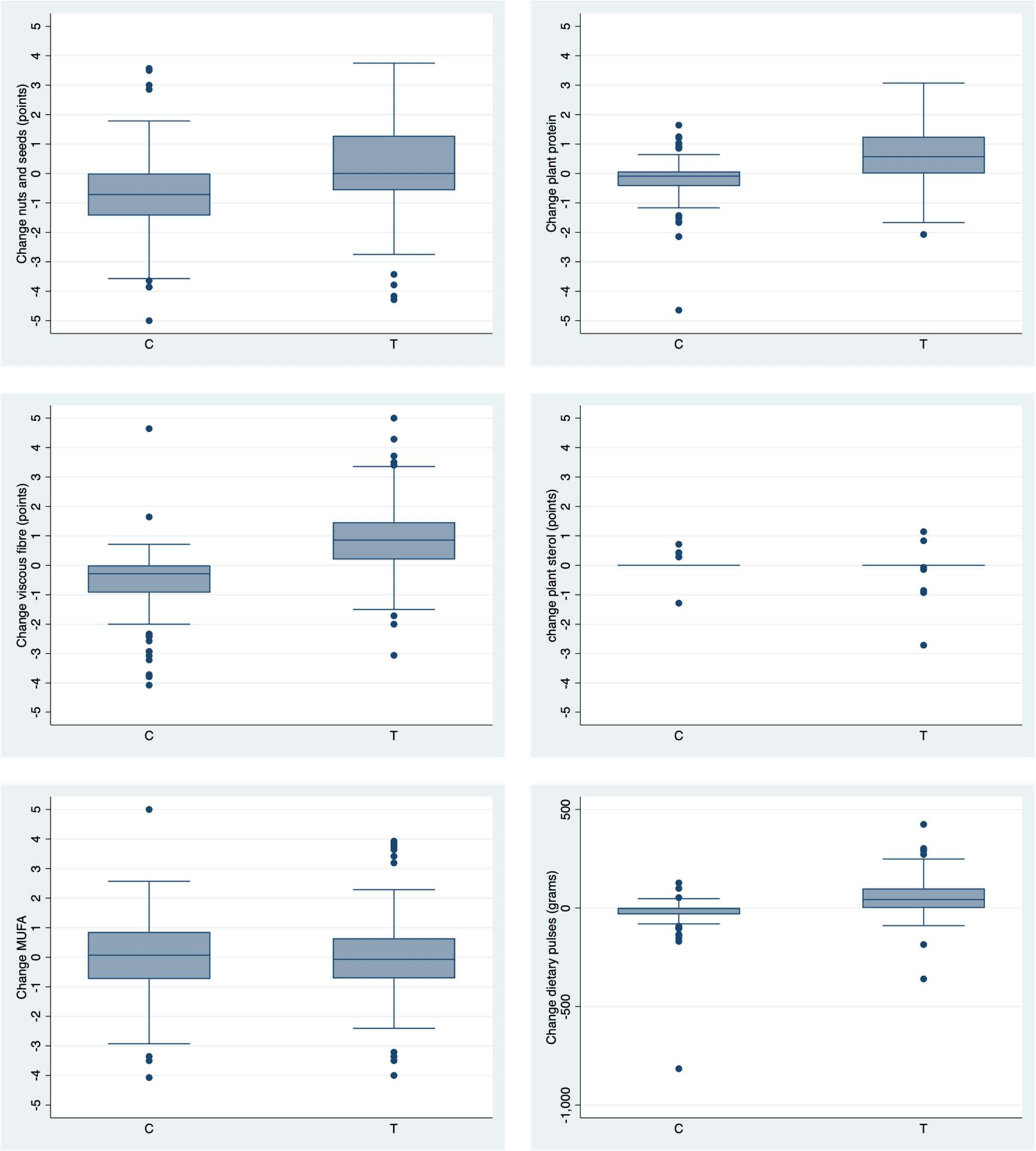

Figure S3: Change in the clinical Portfolio Diet Score by pillars shown by the control (high fibre dietary advice) and test (low glycemic index dietary advice) groups.

Abbreviations: C, control; c-PDS, clinical Portfolio Diet Score; MUFA, monounsaturated fatty acid; T, test.

The center line in each box represents the 50th percentile (median change). The bottom of each box represents the 25th percentile and the top of each box represents the 75th percentile. The whiskers represent the upper and lower extremes with dots showing outliers. All are presented as change in c-PDS points except dietary pulses (grams).

Supplement

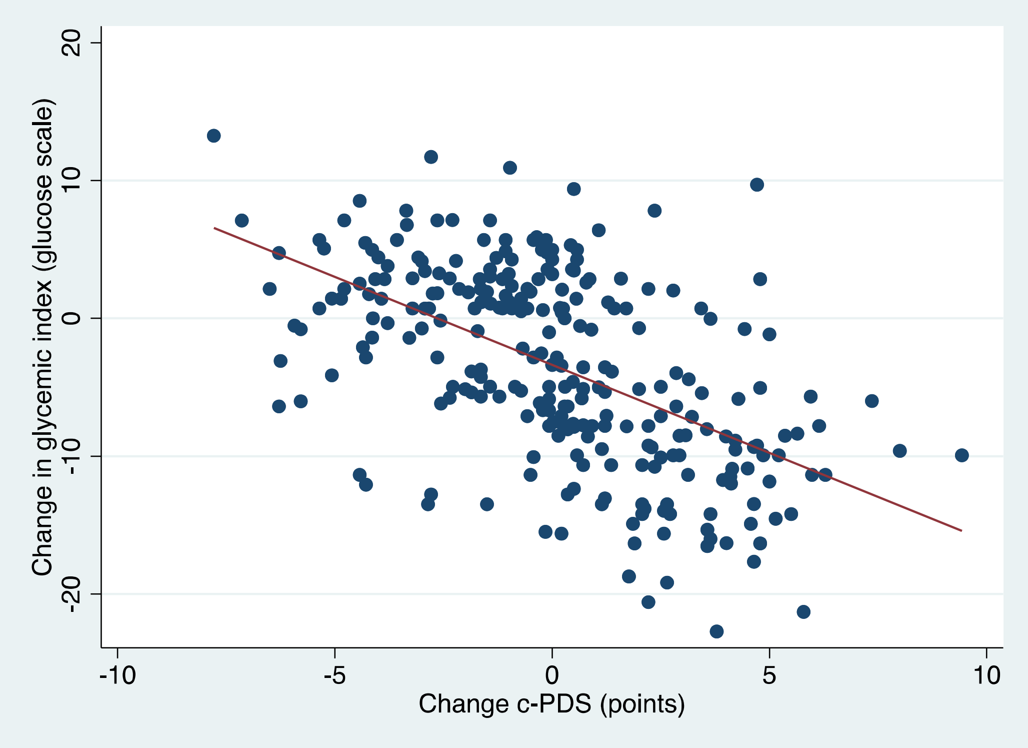

Figure S4: Change in clinical-Portfolio Diet Score verses change in the dietary glycemic index.

Abbreviations: c-PDS, clinical Portfolio Diet Score.

r=-0.55 per c-PDS point, P< 0.001

Abbreviations: c-PDS, clinical Portfolio Diet Score; GI, glycemic index.

GI is presented on the glucose scale, where the GI of glucose is 100.
